# The relation between rice consumption, arsenic contamination, and prevalence of diabetes in South Asia

**DOI:** 10.17179/excli2017-222

**Published:** 2017-10-09

**Authors:** Fatima Ismail Hassan, Kamal Niaz, Fazlullah Khan, Faheem Maqbool, Mohammad Abdollahi

**Affiliations:** 1 Toxicology and Diseases Group, Pharmaceutical Sciences Research Group, Tehran University of Medical Sciences, Tehran, Iran; 2 International Campus, Tehran University of Medical Sciences, Tehran, Iran; 3 Department of Toxicology and Pharmacology, Faculty of Pharmacy, Tehran University of Medical Sciences, Tehran, Iran

**Keywords:** rice, arsenic, diabetes, review

## Abstract

Rice is the major staple food for about two billion people living in Asia. It has been reported to contain considerable amount of inorganic arsenic which is toxic to pancreatic beta cells and disrupt glucose homeostasis. Articles and conference papers published between 1992 and 2017, indexed in Scopus, PubMed, EMBASE, Google, and Google scholar were used. Arsenic exposure has been associated with increased blood glucose and insulin levels, or decreased sensitization of insulin cells to glucose uptake. Several studies have shown the association between inorganic arsenic exposure and incidence of diabetes mellitus. Considerable amounts of arsenic have been reported in different types of rice which may be affected by cultivation methods, processing, and country of production. Use of certain microbes, fertilizers, and enzymes may reduce arsenic uptake or accumulation in rice, which may reduce its risk of toxicity. Combined exposure to contaminated rice, other foods and drinking water may increase the risk of diabetes in these countries. Maximum tolerated daily intake of arsenic contaminated rice (2.1 µg/day kg body weight) has been set by WHO, which may be exceeded depending on its content in rice and amount consumed. Hence, increased prevalence of diabetes in South Asia may be related to the consumption of arsenic contaminated rice depending on its content in the rice and daily amount consumed. In this review, we have focused on the possible relation between rice consumption, arsenic contamination, and prevalence of diabetes in South Asia.

## Introduction

Rice is the major staple food for about two billion people living in Asia (Baldwin et al., 2012[8]). South Asia consists of rapidly growing countries such as India, Bangladesh, Pakistan, Afghanistan, Sri Lanka, Nepal, Maldives, and Bhutan; with the highest population of about 1.2 billion in India and least of about 320,000 in Maldives (Kaplan, 2010[42]; Kohei, 2010[44]). Arsenic has been reported to be present in underground water which is a major source of drinking water in these countries (Brammer and Ravenscroft, 2009[12]). It is also present in rice in different forms and concentrations, which may be affected by cultivation, cooking, and irrigation methods, as well as fertilizers (Lin et al., 2017[48]; Misbahuddin, 2003[58]; Rahman and Hagesawa, 2011[69]; Jayasumana et al., 2015[39]). Other foods that contain inorganic arsenic and may increase the risk of toxicity include seafood, fruits, and vegetables (Saha et al., 1999[75]; Schoof et al., 1999[76]; Shrestha et al., 2003[81]; Das et al., 2004[18]; Munoz et al., 2005[61]; Hossain, 2006[32]; Bhattacharya et al., 2010[10]). Rice together with other food and drinking water may be a source of considerable combined exposure to inorganic arsenic (Marin et al., 1993[53]; Guo et al., 1998[26]; Ahmad, 2001[2]; Mukherjee et al., 2006[60]). Other sources of exposure of most South Asian countries to arsenic aside food include burning coal, pesticides, and illicit liquor (Ahmad, 2001[2]; Chakraborti et al., 2001[13]; Bahadar et al., 2014[7]). Inorganic arsenic which is more toxic is known to cause several diseases and cancers of different parts of the body including the skin, cardiovascular system, and reproductive systems (Huang et al., 2015[34]) and even diabetes through epigenetic mechanisms (Khan et al., 2017[43]). The aim of this review is to determine the possible relation between rice consumption, arsenic contamination, and diabetes in South Asia.

## Methods

This review relied on both review and research articles, and conference papers published between 1992 and 2017, indexed in Scopus, PubMed, EMBASE, Google, and Google scholar. Search terms used were 'South Asia”, 'Rice consumption in South Asia', 'Arsenic contamination', 'Arsenic in Rice', 'Arsenic and Diabetes'. The search yielded *in vitro* and *in vivo* animal and human studies mainly from Asian countries, such as India, Bangladesh, Pakistan, and China, and few from United States. Articles on general studies on effects of chemicals or metals on diabetes or other diseases were excluded. A total of 99 articles were cited.

## Effects of Arsenic on Insulin and Glucose Homeostasis

Diabetes Mellitus (DM) may occur as a result of insufficient insulin production, which may be due to pancreatic disease or injury or inadequate utilization of insulin produced by the body (Njolstad et al., 2003[64]). The major cause of type 1 diabetes mellitus (T1DM) is auto-immune reaction to the proteins produced by pancreatic islet cells, while type 2 diabetes mellitus (T2DM) may be a result of resistance to insulin, impaired secretion, as well as lifestyle changes (Holt, 2004[31]; Chan et al., 2009[14]). Arsenic has been reported to cause injury to the pancreatic beta-cells and apoptosis, which may alter insulin production, function and may result in insulin dependent diabetes mellitus (IDDM) (Johnson and Luciani, 2010[40]). IDDM also known as T1DM is usually diagnosed in childhood, not associated with weight gain but cannot be controlled without insulin administration; while T2DM occur in adults and mostly accompany weight gain, but treatment does not depend solely on insulin (Tabatabaei-Malazy et al., 2016[85]; Alberti and Zimmet,1998[3]). Drugs other than insulin can be used in the treatment of T2DM.

## Possible Mechanisms of Arsenic Induced Hyperglycemia

**Figure 1:**
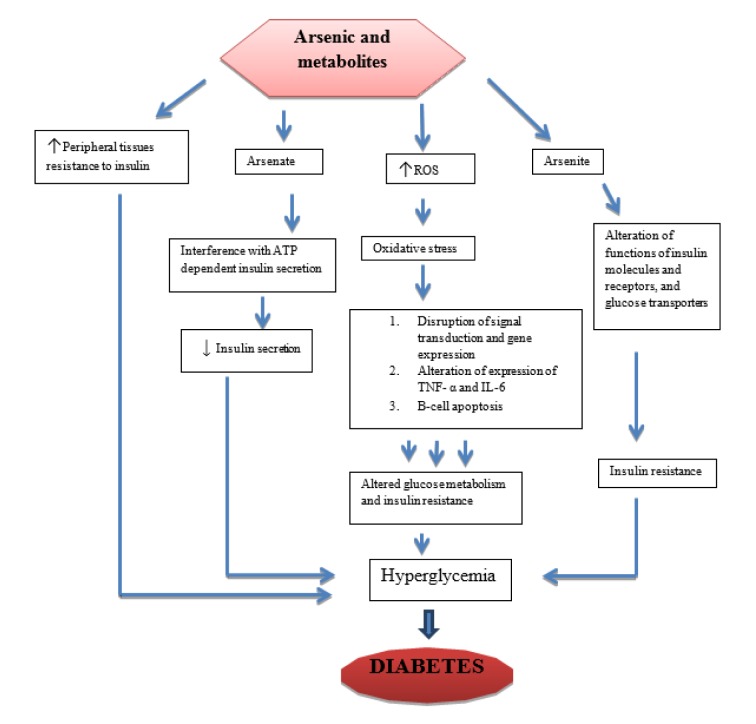
Possible mechanisms of arsenic-induced diabetes

Glucose metabolism can be affected by arsenic and its metabolites (Figure 1(Fig. 1)). Arsenite can bind covalently with sulfhydryl groups in insulin molecules and receptors, enzymes such as pyruvate dehydrogenase and alpha keto-glutarate dehydrogenase, and glucose transporters (GLU-T), which may result in insulin resistance (Tseng, 2004[87]; Fröjdö et al., 2009[25]). Arsenate is the salt or ester of arsenic acid which contain AsO3-4 ion can affect insulin secretion via adenosine triphosphate (ATP) pathway by substituting phosphate in the synthesis of ATP and other phosphate substrates necessary for glucose metabolism (Tseng, 2004[87]). Arsenic and metabolites have also been shown to increase peripheral tissues' resistance to insulin, which might lead to hyperglycemia and subsequent diabetes (Frayn, 2001[24]; Patel and Kalia, 2010[66]). Studies conducted using animal models, both *in vivo* and *in vitro* on high concentration of inorganic arsenic have shown increased blood glucose and insulin levels, while in some cases decreased sensitization of insulin cells to glucose uptake was observed (Walton et al., 2004[89]; Izquierdo-Vega et al., 2006[37]). Transcription factors involved in insulin signal transduction and sensitivity were also altered *in vitro* (Paul et al., 2007[67]). A number of conducted studies have indicated a strong correlation between arsenic exposure and T2DM. One of such studies was carried out on normoglycemic and ovariectomized female mice treated with arsenic trioxide 0.5 ppm, which revealed high blood glucose levels, low plasma insulin, and impaired glucose tolerance, but there was no evidence of insulin resistance (Chen et al., 1992[16]). Insulin resistance is a pathological condition in which cells are normally suppressed to respond to insulin when needed. A significant reduction was also observed in glucose-stimulated insulin secretion in islets of Langerhans isolated from ovariectomized mice treated with arsenic. In another study, exposure to inorganic arsenic in normoglycemic mice indicated pancreatic beta-cell dysfunction, increased gluconeogenesis and oxidative stress in the liver, while in diabetic mice; it impaired glucose tolerance (Liu et al., 2014[50]). Another study carried out in inorganic arsenic highly affected areas on pregnant women indicated that when blood samples were used to determine OGTT, arsenic exposure caused impaired OGTT especially in the third trimester, which may increase the risk of gestational diabetes (Ettinger et al., 2009[22]). Furthermore, to determine the effect of chronic arsenic exposure on pancreatic beta-cells function and insulin sensitivity, serum glucose and insulin concentration values from blood samples of human subjects were used (Díaz-Villaseñor et al., 2013[20]). The results also indicated that chronic inorganic exposure is associated with increased risk of beta-cells dysfunction. Studies conducted in both animals and humans have shown a strong association between chronic inorganic arsenic exposure to the increased risk or prevalence of especially T2DM (Del Razo et al., 2011[19]; Huang et al., 2011[33]; Jovanovic et al., 2013[41]). The recent review indicated that low to moderate concentrations of inorganic arsenic is linked with interaction to components of the epigenome and consequently alterations in glucose transport and/or metabolism and insulin expression/secretion leading to diabetes (Khan et al., 2017[43]).

## Prevalence of Arsenic Exposure and Diabetes in South Asian Countries

Arsenic is a highly dangerous metal found throughout nature; it is present in water, soil and the atmosphere and exposure routes may be via ingestion (most common), inhalation and absorption through the skin (Ng et al., 2001[62]). Arsenic has both cancerous and non-cancerous effects ranging from skin, lung, gastrointestinal tract, bladder cancer, cardiovascular and degenerative diseases, and diabetes, respectively (Khan et al., 2017[43]; Saha et al., 1999[75]; Hodjat et al., 2017[30]). Exposure to arsenic is believed to be majorly by drinking water, and is prevalent in Asian countries such as Bangladesh, China, Taiwan, India, and also Nepal (Marin et al., 1993[53]; Wang et al., 2000[90]; Tandukar and Neku, 2002[86]). Other sources of exposure to arsenic may be from foods contaminated as a result of burning or heating coal and crop drying (Liu et al., 2002[49]). Arsenic can also be found in insecticides, rodenticides, arsenic contaminated beer (Herce-Pagliai et al., 2002[29]), which may lead to inflammation or damage of the liver. It occurs in both organic and inorganic forms with the former being less toxic and latter extremely toxic (Lewis, 2007[46]; Mostafalou and Abdollahi, 2017[59]). A recent study conducted in arsenic contaminated areas in India, showed a greater risk of arsenic toxicity from exposure in children below puberty age. This may be a result of high level of water consumption from these age groups due to their body surface area (Rahman et al., 2001[72]).

**Figure 2:**
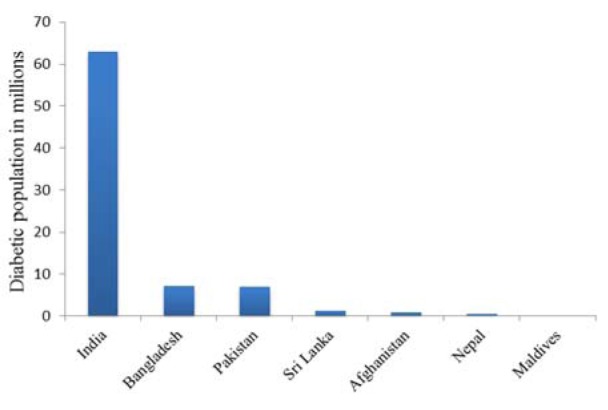
Prevalence of diabetes in South Asian Countries, 2015 International Diabetes Federation (IDF)

South Asian countries seem to have a high incidence of diabetes due to their apparent predisposition for the disease and life style, especially if someone considers the differences between urban and rural populations (Unnikrishnan et al., 2014[88]). Recent studies have shown that the prevalence of DM has been twice as high in the urban areas, especially in India since the early 1970s (Gupta and Misra, 2007[27]; Anjana et al., 2011[5]; Sen et al., 2015[77]). Diabetes occurs in these populations irrespective of age or body mass index which is related to insulin resistance, positive family history, autoimmune reactions, and metabolic disorders (Ng et al., 2001[62]; Ramachandran et al., 2003[74]). Gestational diabetes, which may increase the risk of developing diabetes in future in Asian women, may also be a contributing factor to the prevalence of the disease (Ferrara, 2007[23]). According to the 2015[35] compilation issued by the International Diabetes Federation (IDF), India, Pakistan and Bangladesh have the largest prevalence of DM (Figure 2(Fig. 2)).

Several studies conducted in South Asian countries have revealed the association between exposure to arsenic from different sources and increased prevalence of type 2 diabetes mellitus (T2DM). Higher incidence of T2DM was reported from a study in Bangladesh in subjects with keratosis which is a symptom of arsenic toxicity (Rahman et al., 1998[68]). Major source of exposure to arsenic in these populations may be drinking water. Smith et al. (2000[82]) revealed that over 60 % of the wells in this region contaminated with arsenic above WHO limit of 10 µg/L (WHO, 2004[91]). Islam et al. (2012[36]) also reported high incidence of T2DM from long term exposure to arsenic in drinking water in Bangladesh. In another study, over 29 % of wells in Nepal were found to be contaminated with arsenic, exposure to which was associated with severe symptoms of arsenicosis; which may also increase the risk of T2DM. Studies conducted in Pakistan have also shown the likely exposure to arsenic from contaminated drinking water, vegetables, and grains, as well as increased incidence of T2DM (Mukherjee et al., 2006[60]; Arain et al., 2009[6]; Al-Othman et al., 2016[4]).

## Arsenic Contamination in Rice

Arsenic is present in rice grain produced by different countries at different concentrations (Williams et al., 2005[93]; Zavala and Duxbury, 2008[97]). An estimate revealed that between 2008 and 2009 the per capita rice consumption was found to be highest in India followed by Bangladesh and the least in Pakistan (Baldwin et al., 2012[8]). The presence of arsenic in rice may contribute to the overall risk of developing T2DM in this population (Meharg, 2004[54]).

Arsenic is known to be present naturally in soil in most parts of the world, and food such as rice, other grains and vegetables grown in industrial areas may contain it at high concentration (Seyfferth et al., 2014[79]). Also rice or crops grown in cotton fields or orchards contaminated with arsenic containing fertilizers may comprise high level of arsenic in them (Jayasumana et al., 2015[39]). A study reported that arsenic concentration and nutritional status of rice grain depend on the type and nature of the soil that grown on (Zavala and Duxbury, 2008[97]). Rice has been observed to retain or take up more arsenic mostly the inorganic form from the soil compared to other food products (Williams et al., 2007[94]). Brown rice is said to contain 80 % more inorganic arsenic than white rice, because of the presence of germ layer in brown rice, which is known to retain a considerable amount of inorganic arsenic (Sun et al., 2008[84]). A study revealed that arsenate, a common form of arsenic, is taken up by plants, mainly via phosphate transporters, while in the soil where rice is grown, the most common form is arsenite and is transported via several mechanisms (Ma et al., 2008[51]). Silicon and arsenite occur simultaneously in plants and both accumulate via influx and efflux (Zhao et al., 2009[98]). The possibility of arsenite being transported to the paddy rice root via aquaporin has also been reported (Meharg and Jardine, 2003[55]). The US Environmental Protection Agency (US EPA) has set a standard for arsenic level in food at 5 parts per billion (5 ppb) (Henke, 2009[28]). A conducted research confirmed the presence of both organic and inorganic arsenic in rice at different concentrations depending on country or continent of production (Meharg et al., 2009[56]). Another study on rice products such as rice syrups especially organic brown rice syrup, baby formulas, and energy shot blocks used by athletes indicated that consuming such products may lead to exposure to high concentration of inorganic arsenic, which may exceed the standard 10 µg/L in drinking water (Jackson et al., 2012[38]). Daily consumption of 0.08 μg.g of arsenic in rice is said to be equivalent to 10 μg/L of arsenic in drinking water. Using inductivity coupled plasma mass spectrometry, concentrations between 0.29-0.51 μg/g of arsenic mainly arsenite/arsenate with dimethyl arsenic acid were present in different types of rice obtained from Bangladesh markets grown at different seasons (Williams et al., 2006[92]). High inorganic arsenic content have been reported in areas where arsenic contaminated water is used for irrigation and cooking as a result of deposition in soil and uptake by rice grain (Rahman and Hasegawa, 2011[69]). Rice cooked with contaminated underground water has been shown to contain more arsenic than raw rice which may be as a result of chelation by rice grain or evaporation during cooking (Misbahuddin, 2003[58]; Rahman et al., 2011[71]). Cooking methods (parboiled/non-parboiled rice) have also been shown to affect arsenic retention (Sengupta et al., 2006[78]; Rahman et al., 2006[70]). The increasing population in India and Bangladesh and little rainfall may contribute to the increased use of underground water contaminated with arsenic for rice cultivation, which may also increase its deposition in topsoil (Brammer and Ravenscroft, 2009[12]). Williams et al. (2005[93]) reported different concentrations of inorganic arsenic in white basmati, brown basmati, and long red rice to be 0.02-0.04, 0.04, and 0.05 µg/g, respectively. They also found that arsenic concentration is higher in Thai and Jasmine rice (0.11-0.51 and 0.11 µg/g) found in Thailand compared to the Indian rice. Pal et al. (2009[65]) reported a significant relationship between the amount of arsenic in rice and its concentration in type of water used for irrigation and soil. The bioavailability of inorganic arsenic in cooked rice after consumption has been reported to be above 90 % (Laparra et al., 2005[45]). Its uptake, retention, and transport in Caco-2 cells determined by the same group and revealed a lowest value of 3.9 % and a highest value of 17.8 % equivalent to consuming 5.7 kg and 1.2 kg of cooked rice containing 4.21±0.09 and 2.29±0.05 µg/g daily, may reach the tolerable daily intake recommended by WHO (2.1 µg/kg/day) (Laparra et al., 2005[45]; Williams et al., 2006[92]). Depending on the rice type, inorganic arsenic may contribute 55-79 % maximum tolerable daily intake of arsenic in a Bangladeshi adult weighing 60 kg, which may exceed 100 % level with high concentrations (Williams et al., 2006[92]; Sun et al., 2008[84]).

## Lessening Arsenic Uptake/Accumulation in Rice

Inorganic arsenic uptake by rice from contaminated underground water or soil has been shown to increase the exposure risk and possible health hazard. Although, rice is a major dietary source, lessening of its consumption may reduce the rate of accumulation.

### Role of microbes 

Studies have reported the possibility of reducing or preventing heavy metals uptake by plants, especially rice by the use of certain microbes (Chibuike and Obiora, 2014[17]; Niaz et al., 2016[63]). Rhobdococcus, Comamonas, Serratia, and Streptomyces species are capable of reducing the activity of arsenic in plant roots (Aafi et al., 2012[1]; Yang et al., 2012[96]). Some bacterial strains such as Brevibaccilus species have been shown to possess genes capable of reducing and oxidizing arsenic. Hence accumulation of arsenic is reduced upon colonization (Banerjee et al., 2013[9]; Mallick et al., 2014[52]). These microbes act by changing metal bioavailability via alteration of soil pH, release of chelators, reduction and oxidation reactions (Rajkumar et al., 2012[73]).

### Cultivation methods 

Another study reported the possibility of decreasing arsenic concentration in rice by growing it under aerobic condition which decreases the rate of transfer of arsenic from the soil to the rice grain (Xu et al., 2008[95], Li et al., 2009[47]). This shows that rice grown under flooded condition contained higher concentration of arsenic. It has also been shown that growing rice grain on raised beds will contain less arsenic compared to the conventional flooding methods (Duxbury et al., 2007[21]). 

### Fertilizers

Some fertilizers such as silicon have been shown to reduce the total amount of arsenic in grain; it significantly influences the speciation of arsenic in rice grain and husk by enhancing methylation (Li et al., 2009[47]). Another study reported the inhibitory effect of silicic acid on arsenic uptake by plant, which implied the ability of silicon to reduce arsenic accumulation in rice (Bodgan and Schenk, 2008[11]). Sulfur has been shown to detoxify arsenic and restrict its translocation to shoots through complexation of arsenite with thiols rich peptides (Zhao et al., 2010[99]). Hence sulfur may decrease accumulation in contaminated environment.

### Others

Arsenic accumulation can also be reduced by other methods. Oryza sativa C-type ATP-binding cassette transporter 1 (OsABCC1) present in the upper nodes of rice plant, has been shown to detoxify and reduce the amount of arsenic, as well as restrict its distribution to the grain by sequestering it in the vacuoles of the phloem companion cells of diffuse vascular bundles directly connected to the grain (Song et al., 2014[83]). Arsenic accumulation may also be reduced by breeding rice cultivars with strong oxygen release characteristics as determined by (Mei et al., 2009[57]). In the study, twenty five rice cultivars to determine the correlation between radial oxygen loss and root porosity on arsenic accumulation in grains and straw were used and results revealed that cultivars with high porosity and rates of radial oxygen loss had better capacity to limit the transfer of arsenic to tissues above the ground. Conversion of arsenate to arsenite is the initial stage in detoxification which involves enzymes known as arsenate reductases. High arsenic content 1 (HAC1) is an arsenate reductase which function to reduce the accumulation of arsenic in the root and transport to the shoot by converting arsenate to arsenite in the outer layer of root, thus facilitating efflux of arsenite back into the soil (Chao et al., 2014[15]). Knockout in genes responsible for regulating these enzymes (OsHAC1;1 or OsHAC1;2) led to decreased conversion of arsenate to arsenite in roots, as well as arsenite efflux to external medium associated with increased arsenic accumulation in shoots (Shi et al., 2016[80]).

## Conclusion

The presence of different forms of arsenic in rice and its products pose a serious public health concern especially in Asian countries. Cultivation and cooking of rice with contaminated underground water has been shown to increase the risk of exposure. Therefore, high production and consumption above the permissible limit set by WHO may increase both risk and prevalence of DM. Arsenic has also been reported to exist in other food such as sea food, juices, vegetables and pesticides, which together with contaminated rice and drinking water may lead to exposure to a very high inorganic arsenic concentration. This may have severe effects on human health especially DM. The possibility of reducing the arsenic content by washing thoroughly has been suggested by consumer reports. Arsenic content in rice can be reduced by decreasing its uptake by rice grain. Several methods have been shown to reduce accumulation of arsenic in both soil and rice plant which may improve food safety. Use of arsenic pesticides should be reduced or eliminated. The quality of drinking water in the affected areas must be improved and farming crops on contaminated lands should be avoided strictly. 

Further studies should be conducted on how to minimize exposure to these toxicants. Food and Drug Administration Agencies must update their standards on daily permissible limit for arsenic in different types of food and food products. It is necessary to investigate the exact mechanism of arsenic-induced toxicity due to the consumption of rice. The effects linked with the toxicity of arsenic such as T2DM need further evaluation in order to verify the underlying epigenetic mechanisms. This will help to design a future strategy to minimize the risk of DM associated with consumption of arsenic either in rice or contaminated water.

## Conflict of interest

Authors declare no conflict of interest.

## Acknowledgement

Authors wish to thank Iran National Science Foundation (INSF).
